# The missing link: electronic health record linkage across species offers opportunities for improving One Health

**DOI:** 10.1093/jamiaopen/ooaf151

**Published:** 2025-11-12

**Authors:** Kathleen R Mullen, Nadia Saklou, Adam Kiehl, Toan C Ong, George Joseph Strecker, Sabrina Toro, Sue VandeWoude, Ian M Brooks, Tracy L Webb, Melissa A Haendel

**Affiliations:** Department of Genetics, University of North Carolina at Chapel Hill, Chapel Hill, NC 27599-7264, United States; Department of Clinical Sciences, Colorado State University, Fort Collins, CO 80523, United States; College of Veterinary Medicine and Biomedical Sciences, Colorado State University, Fort Collins, CO 80523, United States; Department of Biomedical Informatics, University of Colorado Anschutz Medical Campus, Aurora, CO 80045, United States; College of Veterinary Medicine and Biomedical Sciences, Colorado State University, Fort Collins, CO 80523, United States; Department of Genetics, University of North Carolina at Chapel Hill, Chapel Hill, NC 27599-7264, United States; Department of Microbiology, Immunology and Pathology, Colorado State University, Fort Collins, CO 80523, United States; Department of Biomedical Informatics, University of Colorado Anschutz Medical Campus, Aurora, CO 80045, United States; Department of Clinical Sciences, Colorado State University, Fort Collins, CO 80523, United States; Department of Genetics, University of North Carolina at Chapel Hill, Chapel Hill, NC 27599-7264, United States

**Keywords:** One Health, electronic health record, data linkage, personally identifiable information, data governance

## Abstract

**Objective:**

Significant opportunities for understanding the co-occurrence of conditions across species in coincident households remain untapped. We determined the feasibility of creating a Companion Care Registry (CCR) for analysis of health data from the University of Colorado Health (UCHealth) patients and their companion animals who received veterinary care at the geographically-adjacent Colorado State University Veterinary Health System (CSU-VHS).

**Materials and Methods:**

Overall populations of the institutions were compared. Using a hybrid deterministic and probabilistic record linkage method, non-medical Personally Identifiable Information was securely matched to determine the total number of UCHealth patients within the HIPAA-compliant Health Data Compass Research Data Warehouse (2015-2024) who took a companion animal to the CSU-VHS (2019-2024).

**Results:**

12 115 matches were identified, indicating 29% of CSU-VHS clients were UCHealth patients.

**Discussion:**

The overlap between CSU-VHS clients and UCHealth patients underscores the potential feasibility and utility of a CCR.

**Conclusion:**

This work provides a mechanism to evaluate environmental and inter-species influences on One Health.

## Background and significance

Accelerating precision medicine within the context of the learning healthcare system requires shifting how we structure, share, and collaboratively analyze data in clinical and translational science. Advances in connecting and integrating information across human health contexts have historically moved relatively slowly. Data from veterinary patients [e.g., veterinary electronic health records (vEHR)] offer an underutilized resource for accelerating translational science and precision medicine. For context, 66% percent or 86.9 million U.S. families own a companion animal, and over 85% of owners consider their pet(s) part of the family.^1^^,^^2^ There is growing awareness of the relevancy of cross-species data, the importance of the human-animal bond, and the need for One Health approaches to health problems. One Health is an international effort that recognizes the interdependence of human, animal, and environmental health. Accordingly, exploring shared veterinary and human health data has the potential to positively impact patient outcomes.^3^

Many disease definitions and proposed pathophysiologies are similar across species, highlighting their potential translational relevance. Because people and their companion animals coexist in the same household (dogs and cats sharing the indoor and/or outdoor environment and horses sharing the outdoor environment with humans), animals may help uncover risk factors, disease pathways, and novel interventions for shared diseases. For example, the body mass index of adult dog owners and dog body condition score were positively correlated in 1 study, where nearly 58% of dog owners and 63% of dogs were overweight or obese,^4^ highlighting the cross-species prevalence and need for successful intervention strategies. Shared behaviors among animal owners and their companion animals suggest that improving the health of 1 species may have indirect health benefits to the other. Dog ownership was reviewed as a solution to promote exercise among adults with prediabetes and Type 2 diabetes (T2D), but more research is needed to fully evaluate the impact of dog ownership on cardiovascular disease risk in patients with T2D.^5^ In addition, the household environment potentially has significant health impacts, such as through exposure to air and water pollution and endocrine disrupting compounds.^6–9^ In one study, dog owners in more disadvantaged neighborhoods reported less on-leash walking activity compared to owners in advantaged neighborhoods,^10^ suggesting that companion animal data can potentially fill significant gaps in understanding health disparities.

## Objective

We hypothesize that a Companion Care Registry (CCR) for geographically overlapping human and veterinary medical institutions would enable investigation of the co-occurrence of conditions and environmental influences in humans and companion animals in coincident households. This project sought to assess the feasibility, in terms of available data, technology, and governance of creating a CCR to assess human and veterinary medical and environmental data at a household level to ultimately improve human and animal patient outcomes.

## Materials and methods

In this feasibility study, de-identified aggregate clinical data from the CSU-VHS vEHR and from the UCHealth Health Data Compass (HDC) research data warehouse (RDW)^11^ at the University of Colorado Anschutz (CUA) were compared to determine similarities and differences between the veterinary and human medical datasets. De-identified aggregate clinical data with cell counts <10 were suppressed.

Additionally, the number of clients of the CSU-VHS who were also patients of UCHealth was determined. Non-medical Personally Identifying Information (PII) from the CSU-VHS client records from years 2019-2024 was linked with patient records in the HDC RDW from years 2015-2024 (the slight variation in the included years was due to a change in the CSU-VHS vEHR in 2019). Exemption status was obtained from the Colorado Multiple Institutional Review Board for the study [“Pilot Project to Assess Overlap between People Receiving Healthcare Locally (UCHealth) and Companion Animal Care at the CSU VHS” (COMIRB # 23-0940)] and deemed not human subjects research by the CSU Institutional Review Board (CSU IRB #1920). A data use agreement was developed and signed by the CSU Board of Governors and the Regents of the University of Colorado for and on behalf of CUA agreeing to the scope and purpose of the project and the obligations and activities of the data recipient, CUA.

PII (first name, last name, street address, city, 5-digit zip code, phone number, and email address) from the CSU-VHS client records was delivered to the Google Cloud-based HDC RDW at CUA via encrypted file transfer to a HIPAA-compliant virtual machine (VM). CSU PII was matched to patient records in the RDW using a hybrid deterministic and probabilistic record linkage method contained in the CU Record Linkage (CURL) Python package (Table 1).^12^

**Table 1.** Deterministic and probabilistic record linkage method.

Method	Linkage variable
Deterministic 1	First6ofFN + First6ofLN + phone
Deterministic 2	First6ofFN + First6ofLN + email
Deterministic 3	First6ofFN + First6ofLN + street_address
Probabilistic	FN, LN, Street address, Phone Blocking scheme 1: FN_Soundex + LN_Soundex Blocking scheme 2: First3ofFN + First3ofLN + state

Abbreviations: FN, first name; LN, last name.

The linkage methods were adopted from the existing method currently used by HDC and applied in a stepwise approach.^12^ Linkages identified using Deterministic Method 1 were removed before Deterministic Method 2 was executed, and linkages identified using Deterministic Method 2 were removed before Deterministic Method 3 was executed (Table 1). Probabilistic Blocking Schemes 1 and 2 were sequentially executed after Deterministic Method 3. The threshold to declare a link was set to 90; therefore, linked pairs with a match score lower than 90 were not considered matches and labeled as “non-matches.”^12^ A weight redistribution method was applied to the probabilistic linkage method in the presence of missing linkage data.^13^ Linkage accuracy of the similar approaches using the same threshold reported in the literature^12^ is Precision = 1.000, Recall = 0.585, *F*-score = 0.738.

PII did not leave the host servers and was deleted after the matching process was complete. Shared data were destroyed after the de-identified aggregate (i.e., total) linked-pair count was obtained. The linked-pair count was used to determine the go/no-go status of the larger CCR project.

## Results

Aggregate data from the CSU-VHS and UCHealth are shown in Table 2. The most common species that visited the CSU-VHS were dogs (48 544), followed by horses (11 781) and cats (9867). Females accounted for 47.9% of the animal patients and 53.4% of the human patients. There were more inpatient visits (16.5% vs 1.0%) and emergency visits (20.7% vs 7.8%) for animals than humans. There were 34 449 (out of 70 192 animals; 49%) animal patients and 2 425 388 (out of 3 234 314 people; 75%) human patients who had >1 healthcare encounter. The median time interval between first and last visits was shorter in animals (149 days) compared to humans (810 days). Venous blood draws were among the procedures common to both animals and humans, suggesting both datasets have robust laboratory measurements.

**Table 2.** Characteristics of the CSU-VHS veterinary and UCHealth human patient populations, respectively, 2019-2024.^a^

	CSU-VHS	UCHealth
Patient species		
Human	NA	3 234 314
Dog	48 544	NA
Horse	11 781	NA
Cat	9867	NA
Patient sex, % female	47.9	53.2
Patient age in years, median		
Human	NA	55
Dog	6.3	NA
Horse	10	NA
Cat	6.7	NA
Visit type (%)^c^		
Inpatient	16.5	1.0
Outpatient	83.5	91.2
Emergency	20.7	7.8
Urgent care	18.7	NA
Patient visits		
# Patients with >1 visit	34 649	2 425 388
Median # of visits (range)	1 (1-100)	<10^b^
Mean # of visits (SD)	2.7 (4.1)	14.6 (30.7)
Time interval in days between first and last visit, median (range) for patients with >1 visit		
	149 (1933)	810 (2191)
Most common procedures (count)		
	Physical examination (198 030)	Blood draw venous (7 128 156)
	Blood draw venous (94 004)	Physical rehab (PT/OT) (2 060 569)
	Complete blood count (71 363)	Electrocardiogram (1 693 227)
	Chemistry panel (56 934)	Manual therapy (1 356 125)
	Blood gas panel (52 404)	Physical therapy (1 338 056)
Most common diagnoses (count)		
	Fit and well (33 074)	Essential (primary) hypertension (1 711 893)
	Malignant lymphoma (17 760)	Unspecified essential hypertension (512 607)
	Malignant neoplasm of skin (12 535)	Hyperlipidemia (498 588)
	Osteosarcoma of bone (10 844)	Type 2 Diabetes (489 919)
	Otitis externa (9492)	Other chronic pain (436 160)
	Atopic dermatitis (9264)	Atherosclerosis (435 056)
	Secondary neoplasm of lymph node (7640)	Hypothyroidism (430 451)
	Periodontal disease (7271)	Nicotine dependence (390 707)
	Rupture of cranial (anterior) cruciate ligament (6972)	GERD (380 818)
	Mitral valve disorder (6542)	Other/unspecified hyperlipidemia (343 962)

a Diagnoses were compared for 2012-2019 for the 2 institutions as coded diagnoses in the CSU-VHS vEHR (added manually) were not available for records post-2019.

b Median patient visits to UCHealth <10 and range in visits not provided because of small cell suppression rules.

c Visit type is treated as a binary in the CSU-VHS vEHR: inpatient or outpatient (emergency and urgent care are separate visit designations within the binary). UCHealth uses ternary designations for visit type.

Abbreviations: CSU-VHS, Colorado State University Veterinary Health System; UCHealth, University of Colorado Health; GERD, gastroesophageal reflux disease; NA, not applicable; PT/OT, physical therapy/occupational therapy; SD, standard deviation.

The most common diagnoses at the CSU-VHS were split between those typically seen in the primary care setting (i.e., fit and well (healthy), periodontal disease, otitis externa, and atopic dermatitis) and those seen in a veterinary referral hospital (i.e., malignant neoplasms, rupture of cranial (anterior) cruciate ligament, and mitral valve disorder).

The most common diagnoses from the UCHealth dataset generally represent chronic diseases (i.e., hypertension, hyperlipidemia, T2D, atherosclerosis, hypothyroidism, chronic pain, and gastroesophageal reflux disorder). Attempts to evaluate comparative drug exposures were limited by non-standard categorization methods.

**Figure 1.**
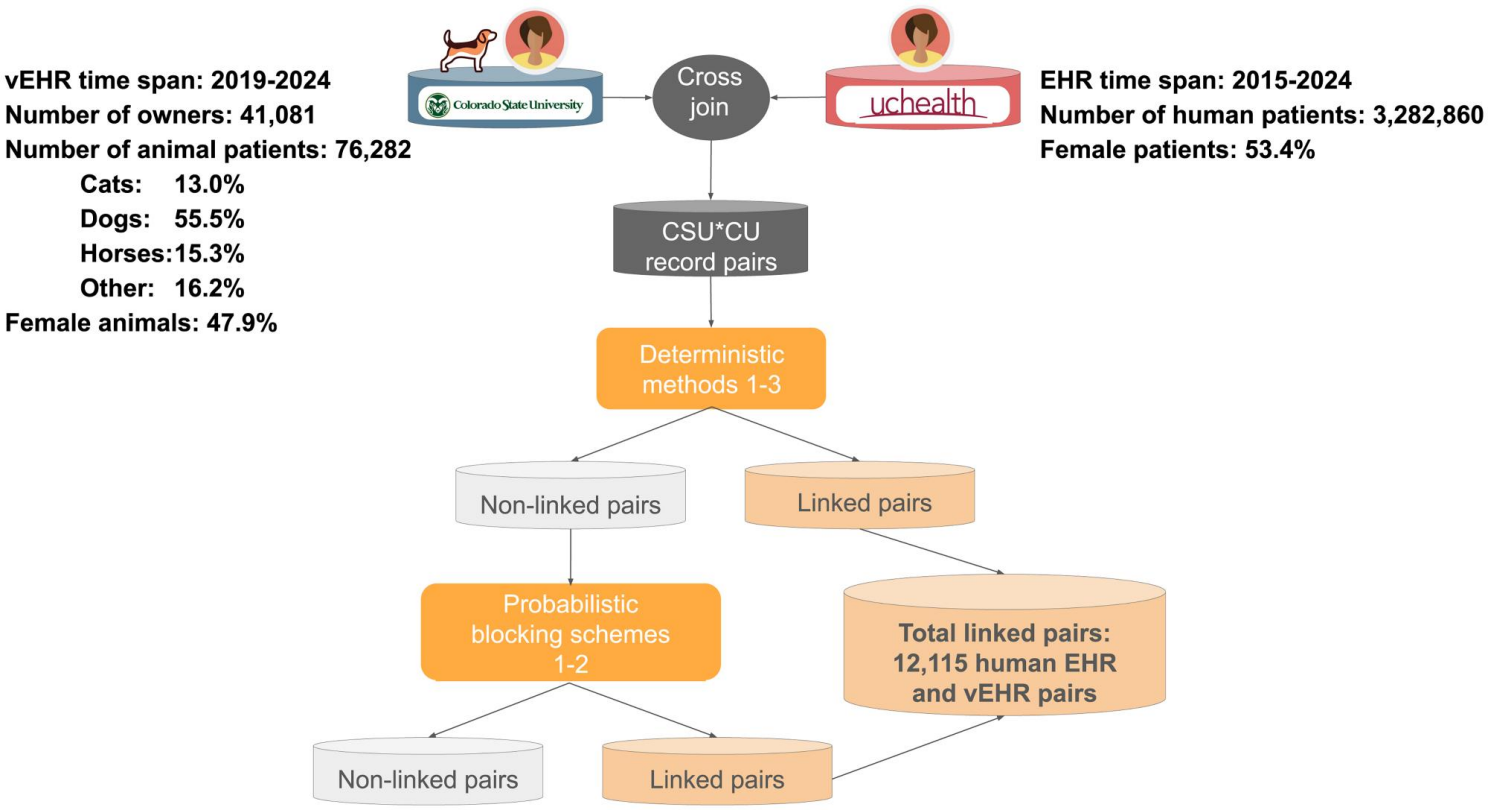
Non-medical PII was linked from geographically adjacent human and veterinary medical academic institutions. The total number of matches (linked pairs of UCHealth patients who took their animal to the CSU-VHS) was 12 115, indicating 29% of companion animal owners were UCHealth patients. CSU-VHS, Colorado State University-Veterinary Health System; EHR, electronic health record; UCHealth, University of Colorado Health; vEHR, veterinary electronic health record.

Securely linking non-medical PII from 41 081 CSU-VHS clients from August 2019 to January 2024 with 3 282 860 UCHealth patients from 2015 to 2024 identified 12 115 matches, indicating that 29% of CSU-VHS clients were UCHealth patients. The overall CSU-VHS client population owned 76 282 animal patients: 55.5% were dogs, 15.3% were horses, 13.0% were cats, and 16.2% were other species (Figure 1).

## Discussion

This pilot study demonstrated the feasibility of developing a CCR utilizing EHR data for geographically overlapping human and veterinary medical institutions. Comparison of the aggregate data across the institutions demonstrated similarities and differences between human and animal health and highlighted areas that need further standardization (e.g., drug categorization). Forty-nine percent of veterinary patients had greater than 1 healthcare encounter compared to 75% of human patients. This difference may be reflective of the CSU-VHS being a referral hospital, and future efforts to connect additional veterinary primary care clinics may be informative for the purpose of creating a longitudinal dataset of linked human-companion animal data. Indeed, limitations of this feasibility study include referral center bias, inability to match data across disparate data sources, lack of linkage validation specific to this study, single-region focus, and small cell-size suppression that may limit analytical capabilities.

Our successful bi-institutional data-sharing and patient matching identified 12 115 client-patient matches between the geographically adjacent hospitals. Considering the favorable results of this study, development of an Observational Medical Outcomes Partnership (OMOP) Common Data Model (CDM)-based^14–17^ CCR for geographically overlapping human and veterinary medical institutions would enable investigation of the co-occurrence of conditions and environmental influences in coincident households. For example, several of the common diagnoses identified in the aggregate data from UCHealth, also occurred frequently in companion animals (e.g., diabetes, hypothyroidism, hypertension, and hyperlipidemia).^18–22^ A CCR would facilitate evaluation of shared risk factors, etiologies, disease course, and treatment needs, options, and responses for these and other conditions. CSU is in the process of transforming its vEHR into the OMOP CDM using custom veterinary-specific concepts and vocabularies where needed.^15^^,^^16^

Efforts to standardize data in vEHR systems using existing terminologies will improve the ability to use veterinary health data in comparative One Health efforts. For example, standardizing drug exposures using RxNorm will resolve the current limited ability to evaluate comparative drug exposures due to non-standard categorization methods.^16^^,^^23^ Integrating open, community-driven, cross-species-enabled ontologies (e.g., from the Monarch Initiative^24^^,^^25^) into the CCR would allow more accurate capture of the rich phenotypic features of conditions and facilitate generalizability and interoperability between humans and veterinary species. Applying open, community-driven ontologies within the CCR will allow for integration of species-specific terms with precise mappings between terms allowing for cross-species comparisons.^26^

Creating the CCR requires strong collaborative efforts between institutions including attention to data governance and data management. Even with strong relationships, the current interinstitutional and interprofessional data sharing and feasibility study took significant time and effort to complete. Shared experiences and established collaborations, such as those created through the National Institutes of Health National Center for Advancing Translational Sciences Clinical and Translational Science Awards (CTSA), and commitment to One Health and interprofessional education efforts enable novel and integrated ways of finding solutions for current multi-species health concerns. For example, in a collaboration between Kansas State University and the University of Missouri, the 1DATA Project led to the development of a Master Sharing Agreement that serves as a template for expediting future One Health research.^27^ In addition, the National COVID Cohort Collaborative (N3C) provides mechanisms and insights into creating a successful governance structure.^28^

Several key steps for successful creation of the CSU-CU CCR and other similar efforts across additional pairs of human and veterinary medical institutions within the CTSA One Health Alliance^29^ can be identified using previous examples.^29^ Obtain institutional review board approval from each institution. Secure inter-institutional data sharing and use agreements. Engage with veterinary stakeholders (pet owners, veterinarians, and veterinary administrators) to ensure transparency and that informed consent collected at admission reserves the right to use data in scientific studies. Ensure safe data transfer and storage from the veterinary hospital to a secure, HIPAA-compliant RDW. Establish a registry review board with members from the medical and veterinary institutions to review registry protocols, data access/use, training requirements, and analysis approvals within a tiered system (aggregate, de-identified, limited datasets). Ensure the appropriateness of results output for dissemination and fair authorship and attribution.

## Conclusion

Data linkage across human and veterinary EHR is a feasible method to explore the dynamics of exposures and diseases shared by people and companion animals. Once fully established and incorporating standardized nomenclature, the CCR will provide a mechanism for future multi-species research studies as well as a blueprint to expand the data linkage efforts to other institutions, enabling investigation into human-animal-environment health interactions on a global scale.

## Data Availability

The data underlying this article are available in the article.
